# Supplementary material for: Multimodal Web-Based Intervention for Cancer-Related Cognitive Impairment in Breast Cancer Patients: Cog-Stim Feasibility Study Protocol
Source: Cancers (Basel). 2021 Sep 28;13(19):4868. doi: 10.3390/cancers13194868 (PMC8508392; doi:10.3390/cancers13194868)
Supplement: Supplementary file 1 [file cancers-13-04868-s001.zip › cancers-1313457-supplementary.pdf]

Supplementary Material

Questionnaire de satisfaction - Patientes

A remplir à la fin de l'intervention

Initiales de la patiente ⌴ ⌴ N° Inclusion ⌴⌴ Date ⌴⌴/⌴⌴/⌴⌴⌴⌴

Questionnaire rempli : oui = 1 non = 0 ⌴

Questionnaire de satisfaction évaluant la perception des patientes atteintes de cancer du sein après avoir suivi le programme APA-Stimulation Cognitive de l'étude Cog-Stim.

- Dans son ensemble, avez-vous **apprécié** le programme ?

  ☐ Oui, beaucoup ☐ Oui, un peu

  ☐ Non, pas vraiment ☐ Non, pas du tout

  → **Si non,** dites-nous pourquoi :

- **Recommanderiez-vous ce programme** ?

  ☐ Oui ☐ Juste les exercices cognitifs

  ☐ Juste l'activité physique adaptée ☐ Non

  → **Si non**, dites-nous pourquoi :

- Avez-vous l'impression que le programme a permis de **réduire vos difficultés cognitives** ?

  ☐ Oui, beaucoup ☐ Oui, un peu

  ☐ Non, pas vraiment ☐ Non, pas du tout

- Avez-vous l'impression que le programme a permis d'améliorer **d'autres aspects de votre qualité de vie** (activités quotidiennes, fatigue, confiance en soi etc.) ?

  ☐ Oui, beaucoup ☐ Oui, un peu

  ☐ Non, pas vraiment ☐ Non, pas du tout

  → **Si oui,** merci de préciser quelles sont les améliorations ressenties :

- Le **nombre de séances** était-il :

  ☐ Très satisfaisant ☐ Satisfaisant

  ☐ Peu satisfaisant ☐ Pas du tout satisfaisant

  → **Si peu ou pas du tout satisfaisant**, dites-nous pourquoi :

- La **durée des séances** était-elle :

  ☐ Très satisfaisante ☐ Satisfaisante

  ☐ Peu satisfaisante ☐ Pas du tout satisfaisante

  → **Si peu ou pas du tout satisfaisante**, dites-nous pourquoi :

- La **difficulté des séances d'activité physique/ l'adaptation à vos besoins spécifiques** était-elle :

  ☐ Très satisfaisante ☐ Satisfaisante

☐ Peu satisfaisante ☐ Pas du tout satisfaisante

→ **Si peu ou pas du tout satisfaisante,** dites-nous pourquoi :

- Le **contenu des exercices cognitifs** était-il :

☐ Très satisfaisant ☐ Satisfaisant

☐ Peu satisfaisant ☐ Pas du tout satisfaisant

→ **Si peu ou pas du tout satisfaisant**, dites-nous pourquoi :

- Le **contenu des séances d'activité physique** était-il :

☐ Très satisfaisant ☐ Satisfaisant

☐ Peu satisfaisant ☐ Pas du tout satisfaisant

→ **Si peu ou pas du tout satisfaisant,** dites-nous pourquoi :

- La **difficulté des exercices cognitifs** était-elle :

☐ Très satisfaisante ☐ Satisfaisante

☐ Peu satisfaisante ☐ Pas du tout satisfaisante

→ **Si peu ou pas du tout satisfaisante**, dites-nous pourquoi :

- **L'utilisation des outils numériques** (plateformes d'entraînement cognitif et d'activité physique adaptée, batterie de tests cognitifs en ligne) était-elle :

☐ Très satisfaisante ☐ Satisfaisante

☐ Peu satisfaisante ☐ Pas du tout satisfaisante

→ **Si peu ou pas du tout satisfaisante,** dites-nous pourquoi (accès et connexion difficiles, navigation difficile sur le site, autre difficulté technique rencontrée) :

- **L'accompagnement par le personnel** (présentation du programme, coach sportif, suivi téléphonique) était-il :

☐ Très satisfaisant ☐ Satisfaisant

☐ Peu satisfaisant ☐ Pas du tout satisfaisant

→ **Si peu ou pas du tout satisfaisant,** dites-nous pourquoi :

- Le **moment** (au cours du parcours de soin) **où ce programme vous a été proposé** était-il :

☐ Très satisfaisant ☐ Satisfaisant

☐ Peu satisfaisant ☐ Pas du tout satisfaisant

→ **Si peu ou pas du tout satisfaisant,** dites-nous pourquoi et à quel moment de votre prise en charge vous le jugeriez plus opportun :

- **Avez-vous des suggestions, des pistes d'améliorations à proposer pour notre programme ?**

Nous vous remercions d'avoir répondu à ce questionnaire
